# Method for the Separation of Titanium, Zirconium, Iron, and Aluminum from One Another and for their Subsequent Determination^1^

**DOI:** 10.6028/jres.064A.056

**Published:** 1960-12-01

**Authors:** Thomas J. Murphy, W. Stanley Clabaugh, Raleigh Gilchrist

## Abstract

A method is described for the separation of titanium, zirconium, iron, and aluminum from one another and for their subsequent determination. Iron is first precipitated with 1-nitroso-2-naphthol after complexing the titanium and zirconium with citrate to prevent their precipitation. Titanium and zirconium are then collectively precipitated with cupferron and subsequently separated from each other by precipitating the titanium with 8-hydroxyquinoline after complexing the zirconium with ethylenediaminetetraacetic acid. Aluminum and zirconium are recovered from their respective solutions by precipitation with cupferron.

The accuracy of the method was proved by analyzing synthetic solutions containing known amounts of the four elements.

## 1. Introduction

This paper describes the separation and determination of titanium, zirconium, iron, and aluminum in barium titanate ceramic dielectrics. These ceramic dielectrics may be dissolved by digestion on the steam bath overnight in a solution containing equal volumes of hydrochloric acid and water. Any undissolved residue may be brought into solution by fusing it with sodium carbonate and treating the resulting fusion with diluted hydrochloric acid. Titanium, zirconium, iron, and aluminum can then be sharply separated from the alkaline earths, the alkalies, and silicon by a double precipitation with 8-hydroxyquinoline.

The procedure described here starts with the collective precipitation of the four elements by 8-hydroxyquinoline. Much of the analytical chemistry involved is new and should have application to analytical problems other than those of ceramic dielectrics.

## 2. Reagents

### Standard Titanium Solution

Fifty milliliters of titanium tetrachloride, purified according to the directions of Clabaugh, Leslie, and Gilchrist [l],^2^ was slowly added to 100 ml of ice cold water. The resulting solution was diluted to 2.5 liters with diluted hydrochloric acid (1+1).^3^ Three 50-g portions of this diluted solution, measured with a weight buret, were each diluted to 250 ml and the titanium content determined by precipitating with cupferron and igniting the precipitate to TiO_2_.

### Standard Iron Solution

About 20 g of ferric chloride hexahydrate, American Chemical Society Reagent grade, was dissolved in 200 ml of diluted hydrochloric acid (1+1). The resulting solution was diluted to 2 liters with water. The titer was determined in a manner similar to that for titanium.

### Standard Zirconium Solution

About 65 g of zirconium sulfate tetrahydrate, purified according to the procedure described by Clabaugh and Gilchrist [2], was dissolved in 1 liter of diluted hydrochloric acid (1+3) and the resulting solution then diluted to 2 liters with diluted hydrochloric acid (1+3). The titer was determined in a manner similar to that for titanium. This titer also includes any hafnium present in the zirconium.

### Standard Aluminum Solution

About 4 g of pure metallic aluminum was dissolved in 400 ml of diluted hydrochloric acid (1+2) and the resulting solution then diluted to 2 liters with water. Three 50-g portions of this diluted solution, measured with a weight buret, were each diluted to 250 ml. To each of these solutions 10 ml of acetic acid was added. The acidity was then reduced to value of *p*H 5.0 with diluted ammonium hydroxide (1+1). The aluminum content was determined by precipitating with 8-hydroxyquinoline and igniting the precipitate to Al_2_O_3_.

### 1-Nitroso-2-Naphthol Reagent Solution

Commercially available samples of 1-nitroso-2-naphthol were found to contain significant amounts of residue on ignition. For three different samples the residues amounted to 0.07 percent, 0.14 percent, and 0.19 percent. Spectrochemical examination of one of the residues showed that it was primarily Fe_2_O_3_, and also contained about 10 percent each of MgO and SiO_2_.

Five grams of 1-nitroso-2-naphthol was placed in a 25-ml porous porcelain filtering crucible and leached with 800 ml of water heated to 60 °C. This treatment dissolved about 0.5 g of the reagent. Five hundread ml of diluted ethanol (1+1), heated to about 60 °C, was then passed through the filter and this dissolved most of the water-washed material. The porcelain filter and residue were air-dried and weighed. The concentration of the reagent in the alcoholic solution was determined by difference. This treatment reduced the residue on ignition from 0.07 to 0.01 percent. *This reagent solution must be freshly prepared since it decomposes on standing.*

### Cupferron Reagent Solution

Five grams of cupferron, the ammonium salt of nitrosophenvlhydroxylamine, was dissolved in 100 ml of water. *This solution must be freshly prepared since it decomposes on standing.*

### Ammonium Acetate Reagent Solution

Five hundred milliliters of acetic acid was cooled in an ice bath and ammonium hydroxide was slowly added to the cold solution until the *p*H of the resulting solution was 7.^4^

### Ethylenediaminetetraacetic Acid

To prepare ethylenediaminetetraacetic acid suitable for use, the practical grade was dissolved in a solution of ammonium hydroxide. This solution was filtered and then neutralized to *p*H 1 with hydrochloric acid to reprecipitate the ethylenediaminetetraacetic acid. The precipitate was caught on a filter, washed well with distilled water, and dried at 100 °C.

### Other Reagents

All the other reagents used conformed to the specifications of the American Chemical Society for reagent chemicals.

## 3. Development of the Procedure

### 3.1. Separation of Iron From Titanium, Zirconium, and Aluminum

Ferric iron is reported to be quantitatively precipitated by 1-nitroso-2-naphthol, (C_10_H_7_O_2_N), in the *p*H range 0.95 to 2.00 [3]. The iron is precipitated as ferric nitrosonaphtholate [4], Fe(C_10_H_6_O_2_N)_3_, which can be ignited to ferric oxide [5], Fe_2_O_3_. Titanium and zirconium are also reported to precipitate with 1-nitroso-2-naphthol from weak acid solutions [6]. Aluminum does not form an insoluble precipitate with 1-nitroso-2-naphthol.

Citrate ion was found to prevent the precipitation of titanium and zirconium nitrosonaphtholates from weakly acid solution, at about *p*H 1.5. Ferric iron was found to precipitate quantitatively from a citrate solution in the *p*H range 1.0 to 2.0. Since aluminum is not precipitated by 1-nitroso-2-naphthol, and since citrate ion prevents the precipitation of titanium and zirconium, the possibility of separating iron from these elements by the precipitation of ferric nitrosonaphtholate from a citrate solution was investigated.

It was found that when precipitations of iron were attempted from solutions having a volume of about 350 ml and containing 500 mg of TiO_2_ and 50 mg of Fe_2_O_3_, or 500 mg of ZrO_2_ and 50 mg of Fe_2_O_3_, or 500 mg of Al_2_O_3_ and 50 mg of Fe_2_O_3_, the results were always high. This was thought to be due to the fact that 1-nitroso-2-naphthol is not very soluble under these conditions and that the ferric nitrosonaphtholate forms so slowly that most of the 1-nitroso-2-naphthol precipitates from solution and adsorbs some of the metallic ions. Ethanol increased the solubility of 1-nitroso-2-naphthol and improved the separation, provided that the ethanol content did not exceed 75 ml of 95-percent ethanol in 350 ml of solution and provided that at least a 0.2-g excess of 1-nitroso-2-naphthol was present. If more ethanol or less 1-nitroso-2-naphthol were present, the precipitation of iron was not complete.

#### a. Separation of Iron From Titanium

Eight synthetic mixtures of iron and titanium were prepared from the respective standard solutions. Each solution contained an amount of titanium equivalent to 500 mg of TiO_2_ and an amount of iron equivalent to from 0.2 mg to 50 mg of Fe_2_O_3_. Ten grams of citric acid and 1.8 g of 8-hydroxyquinoline were added to each solution. The citric acid was added to complex the titanium. The 8-hydroxyquinoline was added to approximate the amount of this reagent that would be present if the mixture was first precipitated with 8-hydroxyquinoline to separate this group of elements from the alkalies and alkaline earths and then taken into solution as described in section 4.1.

The acidity of each solution was adjusted to *p*H 1.5 with diluted ammonium hydroxide (1+1), and then the iron was precipitated with 1-nitroso-2-naphthol reagent solution as described in section 4.2.

The results of these separations are given in table 1.

Chemical analysis of 50 mg of Fe_2_O_3_, separated from 500 mg of TiO_2_, showed that the Fe_2_O_3_ contained slightly more than 0.1 mg, but less than 0.2 mg of TiO_2_.

#### b. Separation of Iron From Zirconium

Five synthetic mixtures of iron and zirconium were prepared from the respective standard solutions. Each solution contained an amount of zirconium equivalent to 500 mg of ZrO_2_ and an amount of iron equivalent to from 0.5 mg to 50 mg of Fe_2_O_3_.

The synthetic mixtures were treated exactly as the iron-titanium mixtures in section a, and the iron in each solution was separated and determined by the method described in section 4.2.

The results of these separations are given in table 2.

Chemical analysis of 50 mg of Fe_2_O_3_, separated from 500 mg of ZrO_2_, showed that the ferric oxide contained about 0.1 mg of ZrO_2_.

#### c. Separation of Iron From Aluminum

Five synthetic mixtures of iron and aluminum were prepared from the respective standard solutions. Each solution contained an amount of aluminum equivalent to 500 mg of Al_2_O_3_ and an amount of iron equivalent to from 0.5 mg to 50 mg of Fe_2_O_3_.

The synthetic mixtures were treated exactly as the iron-titanium mixtures in section a, and the iron in each solution was separated and determined by the method described in section 4.2.

The results of these separations are given in table 3.

Chemical analysis of 50 mg of Fe_2_O_3_, separated from 500 mg of Al_2_O_3_, showed that the ferric oxide contained about 0.1 mg of Al_2_O_3_.

### 3.2. Separation of Titanium and Zirconium From Aluminum

Titanium and zirconium are reported to be quantitatively separated from aluminum by precipitation with cupferron from strongly acidic solution [7]. Titanium is precipitated as titanium cupferrate [8], Ti(C_6_H_5_N_2_O_2_)_4_, which can be ignited to TiO_2_. The literature does not report the formula of the zirconium cupferrate, but, by analogy, it is probably Zr(C_6_H_5_N_2_O_2_)_4_, which can be ignited to zirconium dioxide [9], ZrO_2_.

Synthetic mixtures containing 50 mg of Al_2_O_3_ and 500 mg of TiO_2_, and synthetic mixtures containing 50 mg of Al_2_O_3_ and 500 mg of ZrO_2_ were prepared from their respective standard solutions and the volumes adjusted to approximately 400 ml. Ten grams of citric acid was added to each solution, the solutions were neutralized to *p*H 1.5 with diluted ammonium hydroxide (1+1), and 50 ml of hydrochloric acid added to each. A sufficient quantity of cupferron reagent solution was added to each solution to precipitate the titanium or zirconium and provide about 10 ml, 0.5 gram of cupferron, in excess. The solutions were allowed to stand in a refrigerator for about 4 hr and were filtered through 12.5-cm papers.^5^ The filters and precipitates were washed with a 0.1-percent solution of cupferron in diluted hydrochloric acid (1+9), then transferred to 150-ml beakers and decomposed by heating with an infrared lamp. The beakers were then placed in an electric furnace at 450° C and maintained at this temperature overnight or until there was no visible evidence of carbon. The beakers were gradually cooled to room temperature, and 30 ml of diluted sulfuric acid (1+1) was added to each. The beakers were covered and heated gently on an electric hot plate until the oxides were dissolved. The solutions were cooled, transferred to 600-ml beakers, and diluted to 400 ml with water.

The titanium or zirconium in each solution was reprecipitated, filtered, and washed as described above. To determine the amount of aluminum that contaminated the first precipitate, the filtrates from the second precipitation of titanium or zirconium were cooled in an ice bath and their acidities adjusted to *p*H 4.0 with ammonium hydroxide. The solutions, after standing overnight in a refrigerator, were filtered through 9-cm papers, and the precipitates were washed with a 0.5-percent aqueous solution of cupferron, ignited in porcelain crucibles, and weighed as Al_2_O_3_. The results, given in table 4, show that a considerable amount of aluminum had precipitated with the first titanium or zirconium cupferrates.

It was found, however, that if slightly less cupferron than the amount necessary to precipitate completely the titanium or zirconium was added, and the solution heated to about 60° C and stirred continually, the precipitated cupferrates changed from a bulky, flocculent form to a dense and compact form. The precipitation of titanium or zirconium was then completed by the slow addition of cupferron reagent solution until a permanent precipitate no longer formed. Finally, the solutions were cooled and an additional 10 ml of the cupferron reagent solution was added to each.

**Figure 1:**
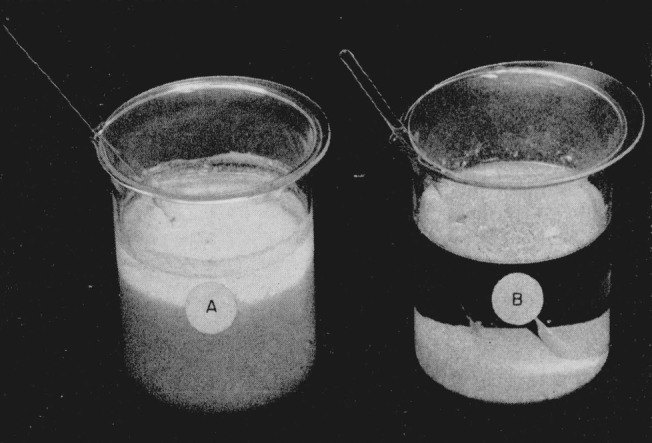
Comparison of the volumes of equal amounts of titanium precipitated with cupferron by the normal method, A, and the revised method, B.

**Figure 2:**
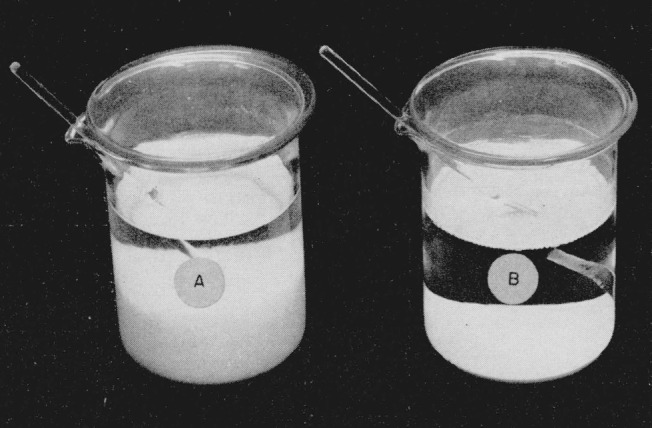
Comparison of the volumes of equal amounts of zirconium precipitated with cupferron by the normal method, A, and the revised method, B.

Figure 1 shows a comparison of the volumes of equal amounts of titanium precipitated with cupferron by the normal method and by the revised method. Figure 2 shows a similar comparison in the case of zirconium. The amounts of titanium and of zirconium involved were equivalent to 500 mg of their respective dioxides.

It was reasoned that since the volumes of the precipitates were much smaller by the revised method of precipitation, a better separation of titanium and zirconium from aluminum might be effected. Synthetic mixtures containing 50 mg of Al_2_O_3_ and 500 mg of TiO_2_, and also mixtures containing 50 mg of Al_2_O_3_ and 500 mg of ZrO_2_, were prepared from the standard solutions. The separations were effected by the revised procedure described above. The results, also reported in table 4, show that the amount of aluminum that was precipitated with the titanium and zirconium was significantly reduced.

### 3.3. Recovery and Determination of Aluminum

Aluminum is reported to be quantitatively precipitated by cupferron from weakly acid solution, probably as Al(C_6_H_5_N_2_O_2_)_3_. This compound can be ignited to [10] Al_2_O_3_.

The completeness of precipitation of aluminum by cupferron from a solution containing large amounts of ammonium chloride and ammonium citrate was determined by the analyses of four synthetic solutions of aluminum containing from 0.5 mg to 50 mg of Al_2_O_3_ which were prepared from the standard solutions. Ten grams of citric acid and 50 ml of hydrochloric acid were added to each solution and the solutions were then diluted to 400 ml. The acidity of each solution was adjusted to *p*H 4.0 with ammonium hydroxide. Enough cupferron reagent solution was added to furnish a 1-g excess of cupferron. The solutions were allowed to stand overnight in a cold box. The precipitates were caught on 12.5-cm papers, washed with a 1-percent solution of cupferron, and ignited to Al_2_O_3_ in porcelain crucibles at 1,100 °C.

The results of these determinations are given in table 5. In each instance the recovery of the added aluminum was complete.

### 3.4. Separation of Titanium From Zirconium

In weakly acid solution, ethylenediaminetetraacetic acid forms a strong mole-to-mole complex with zirconium [11] and a weaker one with titanium [12]. Sen Sarma and Mallik [13] state that the zirconium complex is strong enough to prevent the precipitation of zirconium by 8-hydroxyquinoline in weakly acid solution. Experiments in our laboratory showed that titanium could be almost completely precipitated as titanyl quinolate, TiO-(C_9_H_6_NO)_2_·2H_2_O, at *p*H 4.5 in the presence of ethylenediaminetetraacetic acid, under certain conditions. A 1-g excess of 8-hydroxyquinoline over the amount necessary to precipitate the titanyl quinolate must be present. The excess of ethylenediaminetetraacetic acid should not be greater than 0.5 g over the amount needed to complex the zirconium. The solution should be cooled in an ice bath before filtering. When these conditions are observed, the amount of titanium remaining in solution was found to be about 0.05 mg as TiO_2_. This solubility of titanium was low enough to regard the precipitation as complete.

The titanyl quinolate precipitate can be decomposed by heat from an infrared lamp and converted to TiO_2_ by ignition in an electric furnace. Berg and Teitelbaum [14] reported that this decomposition should be done under a layer of oxalic acid, because of the volatility of titanyl quinolate. Arend and Schnellenbach [15] used a direct decomposition and ignition of titanyl quinolate without the addition of oxalic acid. Experiments in our laboratory showed that when the decomposition was performed with heat from an infrared lamp, no titanyl quinolate was volatilized. Known amounts of titanium were precipitated by 8-hydroxyquinoline, decomposed by heat from an infrared lamp, and ignited to TiO_2_ without any loss of titanium. No trace of titanium was found when the decomposition products from heating titanyl quinolate with an infrared lamp were passed through a solution of sulfuric acid.

Synthetic solutions containing from 475 mg of TiO_2_ and 25 mg of ZrO_2_ to 100 mg of TiO_2_ and 400 mg of ZrO_2_ were prepared from the standard solutions. These mixtures were converted to sulfates by the addition of 15 ml of sulfuric acid and subsequent evaporation to fumes of sulfuric acid. Each solution was then treated as described in section 4.5. Each precipitate of titanyl quinolate was dissolved in 100 ml of diluted sulfuric acid (15+85) and the titanium reprecipitated. To determine the contamination of the first precipitate by zirconium, the filtrates from the second precipitation were made acid with hydrochloric acid and the zirconium recovered by precipitation with cupferron. The solutions were allowed to stand for about 4 hr and filtered through 9-cm papers. The filters and precipitates were washed with a solution of cupferron, transferred to porcelain crucibles, decomposed by an infrared lamp, and ignited to ZrO_2_ at 1000 °C.

The degree of contamination of the titanyl quinolate by zirconium, over the range studied, is given in table 6.

The separation was satisfactory up to the concentration range of about 200 mg of TiO_2_ and 300 mg of ZrO_2_. For amounts of zirconium in excess of this amount a double precipitation should be made, but this increases the amount of soluble titanium weighed with the ZrO_2_ to 0.1 mg of TiO_2_.

Eight synthetic mixtures of titanium and zirconium were prepared from the standard solutions. Fifteen milliliters of sulfuric acid was added to each and the solutions were evaporated to fumes of sulfuric acid. The titanium was then separated and determined by the procedure described in section 4.5. The filtrates were reserved for the determination of zirconium according to the directions in section 3.5. The results of these separations and determinations are given in table 7.

### 3.5. Recovery and Determination of Zirconium

Majumdar and Chowdhury [16] reported that, in the presence of ethylenediaminetetraacetic acid, zirconium can be quantitatively precipitated by cupferron from hydrochloric acid solution.

To confirm this observation, five solutions containing known amounts of zirconium in the range of 0.5 mg to 55 mg of ZrO_2_ were prepared. Fifteen milliliters of sulfuric acid was added to each solution and enough ethylenediaminetetraacetic acid to furnish a 0.5-g excess over the amount necessary to complex the zirconium. The acidity of each solution was adjusted to *p*H 1.5 with ammonium hydroxide, and then to *p*H 4.5 with the ammonium acetate reagent solution. Fifty milliliters of hydrochloric acid was added to each solution and enough cupferron to furnish an excess of 1 g. The solutions were allowed to stand overnight in a cold box, then filtered through 12.5-cm papers. The filters and precipitates were decomposed in porcelain crucibles, and ignited to ZrO_2_.

The results are given in table 8.

The filtrates obtained according to the procedure in section 3.4 were acidified with 50 ml of hydrochloric acid and the zirconium recovered and determined by the above procedure. These results are given m table 7.

## 4. Procedure for Separating Titanium, Zirconium, Iron, and Aluminum From One Another

The following procedure is designed for a total quantity of about 0.5 g of mixed oxides. The maximum amount of each oxide that can be present is: 0.5 g of TiO_2_; 0.25 g of ZrO_2_; 0.05 g of Fe_2_O_3_; and 0.05 g of Al_2_O_3_. The minimum amount of ZrO_2_, Fe_2_O_3_, and Al_2_O_3_ that can be determined is about 0.0005 g each. A very accurate determination of these elements requires a knowledge of the approximate composition of the sample.

### 4.1. Preparation of the Solution for Analysis

Synthetic solutions were prepared by mixing weighed portions of each of the four standard solutions so that the above maxima and minima were observed. Each of the synthetic solutions was treated as follows:

Dilute the synthetic solution to about 250 ml. To precipitate the metals as their respective quinolates, add 20 ml of acetic acid containing sufficient 8-hydroxyquinoline to provide approximately 1 g in excess of the amount required to precipitate all four metals. The nominal compositions of the metal quinolates are: TiO(C_9_H_6_NO)_2_; Zr(C_9_H_6_NO)_4_; Fe(C_9_H_6_NO)_3_; and Al(C_9_H_6_NO)_3_.

Neutralize the solution to an acidity of *p*H 5 by adding diluted ammonium hydroxide (1+1). Wash the electrodes of the *p*H meter with water and wipe them with, bits of filter paper. Reserve these bits of paper and later add them to the mixed quinolate precipitate.

Digest the solution containing the mixed quinolate precipitate for a few hours on the steam bath and then cool it to room temperature. Filter the solution through a 12.5-cm paper and wash the precipitate and filter with a solution of water that has been saturated with 8-hydroxyquinoline.

Dissolve the mixed quinolates of titanium zirconium, iron, and aluminum in the following manner. Place the filter and precipitate in the beaker in which the precipitation was made and add 100 ml of a 10-percent solution of citric acid in diluted hydrochloric acid (5+95) and digest on the steam bath until solution of the precipitate is effected. Break up the filter paper by stirring the solution. Filter the solution through an 11-cm paper and wash the filter with a hot (70 °C) 2-percent solution of citric acid in diluted hydrochloric acid (1+99). Next, wash it with a hot (70 °C) solution of ammonium citrate, prepared by dissolving 10 g of citric acid in 400 ml of water, adjusting the alkalinity to between *p*H 8.5 and 9.0 with ammonium hydroxide, and finally diluting to a volume of 500 ml.^6^

### 4.2. Separation of Iron From Titanium, Zirconium, and Aluminum and its Subsequent Determination

Evaporate the solution obtained as described above to about 150 ml. Add 10 ml of hydrochloric acid and adjust the acidity of the solution to *p*H 1.5 with diluted ammonium hydroxide (1+3). Digest the solution on a steam hath for one-half hour. Add enough ethanol so that the final solution will contain 75 ml of ethanol after the addition of the 1-nitroso-2-naphthol reagent solution which contains 50 percent of ethanol by volume. Slowly add the 1-nitroso-2-naphthol reagent solution to the warm solution until the approximate amount necessary to precipitate the iron as Fe(C_10_H_6_O_2_N)_3_ has been added and/allow the solution to stand at room temperature for one-half hour. Determine the *p*H of the solution and adjust, if necessary, to *p*H 1.5 with diluted hydrochloric acid (1+9). Add enough 1-nitroso-2-naphthol solution to produce a 0.2-g excess of the reagent and allow the solution to stand overnight. Filter the solution through a 9-cm paper and thoroughly wash the precipitate and filter with a warm (60 °C) solution of ammonium citrate. The wash solution is prepared by dissolving 10 g of citric acid in 400 ml of water, adjusting the acidity to *p*H 4.5 with ammonium hydroxide, and diluting to 500 ml.

Decompose the precipitate and filter in a porcelain crucible by heat from an infrared lamp. Complete the ignition at 900 °C in an electric furnace, and weigh the residue as Fe_2_O_3_.

### 4.3. Separation of Titanium and Zirconium From Aluminum

Add 50 ml of hydrochloric acid to the filtrate obtained in the separation of iron by 1-nitroso-2-naphthol. Evaporate the solution to about 200 ml on a steam bath to free it of ethanol. Cool the resulting solution to about 60 °C and slowly add enough cupferron reagent solution to precipitate only about 90 percent of the titanium and zirconium. Digest the solution at this temperature and stir the solution until the precipitate changes from a bulky, flocculent form to one of dense compact nature. Then, slowly add cupferron reagent solution until a permanent precipitate no longer forms. Cool the solution to room temperature and add 10 ml more of the cupferron reagent solution. Allow the solution to stand about 4 hr in a refrigerator. Catch the precipitate on a 12.5-cm paper, and wash it with a 0.1-percent solution of cupferron in diluted hydrochloric acid (1+9).

Reserve the filtrate for the determination of aluminum as described in section 4.4. Reserve the precipitate for the separation of titanium from zirconium as described in section 4.5.

### 4.4. Recovery and Determination of Aluminum

Cool the filtrate obtained in the separation of titanium and zirconium from aluminum (section 4.3) in an ice-bath and neutralize it to *p*H 4.0 with diluted ammonium hydroxide (1+1). Add 20 ml of the cupferron reagent solution and allow the solution to stand overnight in a refrigerator. Catch the precipitate on a 12.5-cm paper and wash it with a 1-percent aqueous solution of cupferron. Place the filter and precipitate in a porcelain crucible and decompose them by heating with an infrared lamp. Complete the ignition in an electric furnace at 1, 100°C. and weigh the residue as Al_2_O_3_.

### 4.5. Separation of Titanium From Zirconium and its Subsequent Determination

Place the precipitate of titanium and zirconium cupferrates obtained in section 4.3 in a 150-ml beaker and decompose it and the filter with heat from an infrared lamp. Then place the beaker and its contents in an electric furnace and raise the temperature to 450 °C. Heat them at this temperature overnight or until carbon is no longer visible. Do not heat above 450 °C because the oxides so formed are difficultly soluble in sulfuric acid. Allow the beaker and its contents to cool to room temperature.

To the beaker containing the mixed titanium and zirconium oxides, add 30 ml of diluted sulfuric acid (1+1). Place the covered beaker on an electric hot plate and dissolve the oxides by heating at a medium heat. Cool the solution, dilute it to 100 ml with water, and filter it through a 9-cm paper, to free the solution of dehydrated silica and to retain any undissolved oxides. To determine the amount of undissolved oxides, wash the paper with diluted sulfuric acid (1+9) and ignite it in a platinum crucible. Treat the ash with 2 ml of hydrofluoric acid and 2 drops of sulfuric acid and heat to volatilize any silica present as silicon tetrafluoride. Ignite the crucible again and weigh any residue as TiO_2_.^7^ If significant, this amount should be added to that determined later.

Add enough solid ethylenediaminetetraacetic acid to the solution to give an excess of 0.5 g over the approximate amount required to complex the zirconium. Then add enough solid 8-hydroxyquinoline to the solution to give an excess of 1 g over the amount required to precipitate the titanium. Zirconium forms a one-to-one molar complex with ethylenediaminetetraacetic acid, and one mole of titanyl ion is precipitated by two moles of 8-hydroxyquinoline. Heat the solution on the steam bath until the solid ethylenediaminetetraacetic acid and solid 8-hydroxyquinoline are dissolved. Neutralize the clear solution to *p*H 1.0 with diluted ammonium hydroxide (1+1) and allow it to stand for 30 min. Dilute it to about 450 ml and add 1-ml portions of the ammonium acetate reagent solution until a permanent precipitate forms or an acidity of pH 2.0 is obtained, whichever occurs first. Heat the solution on the steam bath until the precipitate settles. Add four 5-ml portions of the ammonium acetate reagent solution at 10-minute intervals. Next heat the solution for about 1 hour longer and then cool it to room temperature. Adjust the acidity of the solution to *p*H 4.5 with the ammonium acetate reagent solution. Heat the solution on the steam bath overnight, cool it in an ice bath for 4 hr, and filter it through a 12.5-cm paper. Wash the precipitate and filter with a solution prepared by dissolving 1 g of 8-hydroxyquinoline in 15 ml of acetic acid, diluting it to 400 ml, adding 0.5 g of ethylenediaminetetraacetic acid, neutralizing the solution to *p*H 4.5 with diluted ammonium hydroxide (1+1), and finally diluting to a volume of 500 ml.

Place the precipitate and filter in a porcelain crucible. Decompose the precipitate and filter by heating with an infrared lamp. Ignite the residue in an electric furnace at 1,000 °C, and weigh it as TiO_2_.

### 4.6. Recovery and Determination of Zirconium

To the filtrate reserved from section 4.5, add 50 ml of hydrochloric acid. Add a sufficient quantity of cupferron reagent solution to precipitate the zirconium and to provide an excess of about 1 g of cupferron. It requires 4 moles of cupferron to precipitate 1 mole of zirconium. Allow the solution and precipitate to stand in a refrigerator overnight. Catch the precipitate on a 12.5-cm paper and wash it with a 0.2-percent solution of cupferron in diluted hydrochloric acid (1+19). Decompose the precipitate and filter in a porcelain crucible by heat from an infrared lamp. Complete the ignition in an electric furnace at 1,000 °C and weigh the residue as ZrO_2_.

## 5. Results

The results obtained when the foregoing procedure was applied to the analysis of synthetic mixtures of titanium, zirconium, iron, and aluminum are given in table 9.

## Figures and Tables

**Table 1:** Results obtained in the separation of iron from titanium by 1-nitroso-2-naphthol

Experiment	Titanium dioxide	Ferric oxide		
	Added	Added	Found	Error
				
	*mg*	*mg*	*mg*	*mg*
1	500	0.2	0.2	0.0
2	500	.5	.5	.0
3	500	2.5	2.6	+.1
4	500	4.1	4.3	+.2
5	500	5.1	5.2	+.1
6	500	11.2	11.3	+.1
7	500	23.4	23.6	+.2
8	500	48.8	48.0	+.1

**Table 2:** Results obtained in the separation of iron from zirconium by 1-nitroso-2-naphthol

Experiment	Zirconium dioxide	Ferric oxide		
	Added	Added	Found	Error
				
	*mg*	*mg*	*mg*	*mg*
1	500	0.5	0.5	0.0
2	500	2.6	2.6	.0
3	500	11.1	11.0	−.1
4	500	24.0	24.1	+.1
5	500	51.4	51.6	+.2

**Table 3:** Results obtained in the separation of iron from aluminum by 1-nitroso-2-naphthol

Experiment	Aluminum oxide	Ferric oxide		
	Added	Added	Found	Error
				
	*mg*	*mg*	*mg*	*mg*
1	500	0.5	0.6	+0.1
2	500	2.5	2.6	+.1
3	500	10.2	10.3	+.1
4	500	24.6	24.5	−.1
5	500	50.5	50.4	−.1

**Table 4:** Comparison of results obtained by varying the conditions of precipitation in the separation of titanium and zirconium from aluminum by cupferron

Experiment	Conditions of precipitation	TiO_2_	ZrO_2_	Al_2_O_3_	Al_2_O_3_ found in cupferron precipitate
		Added	Added	Added	
					
		*mg*	*mg*	*mg*	*mg*
1	Normal	500	…………	50	1.0
2	Revised	500	…………	50	0.1
3	Normal	…………	500	50	.3
4	Revised	…………	500	50	.0

**Table 5:** Recovery of aluminum by precipitation with cupferron

Experiment	Aluminum Oxide		
	Added	Found	Error
			
	*mg*	*mg*	*mg*
1	0.5	0.5	0.0
2	4.8	4.7	−.1
3	23.4	23.3	−.1
4	51.5	51.7	+.2

**Table 6:** Experiments which show the degree of contamination of titanium quinolate by zirconium

Experiment	TiO_2_	ZrO_2_	ZrO_2_ found in Ti precipitate
	Added	Added	
			
	*mg*	*mg*	*mg*
1	475	25	0.0
2	450	50	.0
3	425	75	.0
4	400	100	.1
5	300	200	.2
6	200	300	.1
7	100	400	.4

**Table 7:** Results obtained in the separation of titanium from zirconium by precipitation with 8-hydroxyquinoline in a solution containing ethylenediaminetetraacetic acid

Experiment No.	Titanium dioxide			Zirconium dioxide		
	Added	Found	Error	Added	Found	Error
						
	*g*	*g*	*mg*	*g*	*g*	*mg*
1	0.5037	0.5037	0.0	0.0005	0.0006	+0.1
2	.4981	.4984	+.3	.0009	.0009	.0
3	.4831	.4830	−.1	.0067	.0066	−.1
4	.4808	.4809	+.1	.0093	.0092	−.1
5	.4605	.4608	+.3	.0268	.0268	.0
6	.4468	.4471	+.3	.0606	.0604	−.2
7	.3941	.3944	+.3	.1035	.1035	.0
8	.2905	.2907	+.2	.2185	.2185	.0

**Table 8:** Recovery of zirconium by precipitation with cupferron in a solution containing ethylenediaminetetraacetic acid

Experiment	Zirconium dioxide		
	Added	Found	Error
			
	*mg*	*mg*	*mg*
1	0.6	0.6	0.0
2	10.3	10.3	.0
3	25.2	25.1	−.1
4	55.1	55.2	+.1

**Table 9:** Results obtained in the sepamtion of titanium, zirconium, iron, and aluminum from one another

Experiment	Titanium dioxide			Zirconium dioxide			Ferric oxide			Aluminum oxide		
	Added	Found	Error	Added	Found	Error	Added	Found	Error	Added	Found	Error
												
	*g*	*g*	*mg*	*g*	*g*	*mg*	*g*	*g*	*mg*	*g*	*g*	*mg*
1	0.4964	0.4962	−0.2	0.0006	0.0007	+0.1	0.0005	0.0006	+0.1	0.0005	0.0007	+0.2
2	.4980	.4979	−.1	.0102	.0102	.0	.0051	.0051	.0	.0047	.0048	+.1
3	.4269	.4269	.0	.0244	.0245	+.1	.0209	.0210	+.1	.0226	.0228	+.2
4	.3476	.3477	+.1	.0544	.0543	−.1	.0479	.0480	+.1	.0522	.0520	−.2
5	.3422	.3424	+.2	.1028	.1027	−.1	.0124	.0122	−.2	.0050	.0051	+.1
6	.3210	.3208	−.2	.1530	.1528	−.2	.0055	.0054	−.1	.0097	.0100	+.3
7	.2829	.2832	+.3	.2064	.2062	−.2	.0105	.0105	.0	.0048	.0049	+.1
8	.2323	.2326	+.3	.2511	.2510	−.1	.0054	.0055	+.1	.0092	.0090	−.2

## Footnotes

2 Figures in brackets indicate the literature references at the end of this paper.

3 When the use of an acid is prescribed with no indication of strength or dilution, the acid to be used is the concentrated acid. Dilutions are indicated by the volumes of acid and water mixed to prepare a diluted reagent. Diluted hydrochloric acid (1+x) means a diluted acid prepared by mixing 1 volume of the concentrated acid with x volumes of distilled water. Similar designations are used for ammonium hydroxide.

4 All *p*H measurements were made with a glass-electrode *p*H meter.

5 All filter paper used was quantitative, ashless filter paper of close, dense texture suitable for retaining the finest precipitates.

6 When filters were ignited after the above treatment, no residue was found.

7 The residue in most cases was found to be less than 0.1 mg. In those cases n which it exceeded 0.1 mg, it was found to be TiO_2_.
